# The Presence, Location, and Degree of Late Gadolinium Enhancement in Relation to Myocardial Dysfunction and Poor Prognosis in Patients with Systemic Lupus Erythematosus

**DOI:** 10.3390/jcdd10110451

**Published:** 2023-10-31

**Authors:** Xiaojin Feng, Peijun Liu, Xiaohang Liu, Tianchen Guo, Xinhao Li, Huaxia Yang, Wei Chen, Yining Wang, Shuyang Zhang

**Affiliations:** 1 Department of Cardiology, Peking Union Medical College Hospital, Chinese Academy of Medical Sciences and Peking Union Medical College, Beijing 100730, China; xiaojinfengtmu@foxmail.com (X.F.); hanghang165@163.com (X.L.); guotianchenjn@163.com (T.G.); 17865192520@163.com (X.L.); chenwei6@medmail.com.cn (W.C.); 2 Department of Radiology, Peking Union Medical College Hospital, Chinese Academy of Medical Sciences and Peking Union Medical College, Beijing 100730, China; liupeijun_yu@163.com; 3 Department of Rheumatology and Immunology, Peking Union Medical College Hospital, Chinese Academy of Medical Sciences and Peking Union Medical College, Beijing 100730, China; yanghuaxia2013@163.com; 4 State Key Laboratory of Complex, Severe, and Rare Diseases, Peking Union Medical College Hospital, Chinese Academy of Medical Science and Peking Union Medical College, Beijing 100730, China

**Keywords:** systemic lupus erythematosus, myocardial fibrosis, late gadolinium enhancement, myocardial work, prognosis

## Abstract

Patients with systemic lupus erythematosus (SLE) typically develop myocardial fibrosis. No studies have investigated the clinical significance of the presence, location, and degree of fibrosis in SLE patients. Seventy-four SLE patients were included. Thirty-seven non-autoimmune disease patients and thirty-seven healthy individuals were included as controls. Myocardial fibrosis was evaluated at cardiac magnetic resonance via a qualitative and quantitative assessment of late gadolinium enhancement (LGE). Myocardial function was measured via speckle-tracking echocardiography. All patients were followed up for the occurrence of major adverse cardiac events (MACE). The presence, locations, and degrees of LGE disturbed regional and global myocardial function. The presence of LGE, left ventricular free-wall LGE (LVFW LGE), and severe LGE were all independent predictors of MACE in SLE patients [LGE presence HR: 3.746 (1.434–9.79), *p* = 0.007; LVFW LGE HR: 2.395 (1.023–5.606), *p* = 0.044; severe LGE HR: 3.739 (1.241–11.266), *p* = 0.019]. LGE combined with SLE-related organ damage identified patients at high risk of MACE (*p* < 0.001). In conclusion, the presence, degree, and location of LGE were associated with myocardial dysfunction. The presence, location, and degree of LGE had the potential to independently predict poor prognosis and improve risk stratification in SLE patients.

## 1. Introduction

Systemic lupus erythematosus (SLE) is an autoimmune disease characterized by the presence of nuclear autoantibodies, which can cause the inflammatory injury of multiple organs, including all components of the cardiovascular system. Accumulating evidence has shown elevated cardiovascular morbidity and mortality in SLE [1]. Myocardial injury is a potentially fatal manifestation of SLE. An important histopathological injury in the myocardium of patients with SLE is myocardial fibrosis. Previous studies have revealed the presence of myocardial fibrosis in 30–70% of patients with SLE [2]. Myocardial fibrosis may increase the risk of heart failure [3] and emerge as a strong predictor of adverse cardiac events and increased mortality [4].

Cardiovascular magnetic resonance is a non-radiating technique that is considered the primary imaging modality for myocardial tissue characterization. The application of late gadolinium enhancement (LGE) is currently the most reliable method for the non-invasive detection of focal myocardial fibrosis [5]. LGE has been applied to facilitate the identification of myocardial fibrosis in various diseases [6], including SLE [7]. Recently, a novel non-invasive left ventricular pressure-strain loop method proposed by Russell et al. has been applied to assess myocardial function [8]. It takes afterload into account by analyzing strain in relation to non-invasive left ventricular pressure and eventually generates myocardial work indices [9]. In addition, peak strain dispersion (PSD) is the standard deviation of the peak time of the longitudinal strain in each segment of the left ventricle, which could accurately reflect the coordination of myocardial movement [10].

The presence of fibrosis is considered to be associated with myocardial dysfunction and poor outcomes in non-ischemic cardiomyopathy [11,12]. Different locations and degrees of fibrosis are linked with adverse cardiac prognosis in cardiomyopathy [4,13,14]. However, whether myocardial fibrosis with different characteristics is associated with myocardial dysfunction in SLE patients has not yet been well-studied. Furthermore, the prognostic significance of myocardial fibrosis remains unknown in patients with SLE. The present study aims to investigate the correlation between the presence, degree, and location of myocardial fibrosis and myocardial dysfunction and explore the prognostic value of myocardial fibrosis with different characteristics in patients with SLE.

## 2. Materials and Methods

### 2.1. Study Population

Between June 2013 and July 2023, a cohort of SLE patients at the Peking Union Medical College Hospital was consecutively enrolled according to the following inclusion criteria: (i) the diagnosis of SLE confirmed according to the American Society of Rheumatology in 1997 [15]; (ii) patients with clinically suspected myocardial involvement who met the indications for echocardiography and cardiovascular magnetic resonance, including the new onset or persistence of cardiac symptoms (chest pain, dyspnea, or palpitation), electrocardiogram abnormalities, or elevated cardiac biomarkers [5]. We excluded patients for the following reasons: (i) age < 18 years; (ii) ischemic coronary disease defined as the presence of epicardial coronary artery diameter stenosis > 70% and severe valvular disease; (iii) an interval of echocardiography and cardiovascular magnetic resonance imaging examinations more than 1 month; and (iv) poor image quality. Patients with non-autoimmune disease-related non-ischemic cardiomyopathy were included as a control group and were matched to the case group for age, sex, and left ventricular ejection fraction. Additionally, age- and sex-matched healthy individuals were recruited and underwent cardiovascular magnetic resonance and echocardiographic examinations. This study was conducted in accordance with the ethical principles for medical research involving human subjects described in the Declaration of Helsinki. Before inclusion in this study, informed consent was obtained from all subjects.

### 2.2. Clinical Data

Demographic data, detailed symptoms, the results of laboratory tests, and medical treatments were collected based on medical records. The assessment of SLE-related disease activity (SLE Disease Activity Index 2000 (SLEDAI-2K)) and organ damage (the Systemic Lupus International Collaborating Clinics/American College of Rheumatology (SLICC/ACR) Damage Index (SDI)) was performed on all patients [16,17]. Laboratory investigations included autoantibodies (anti-nuclear antibodies, antiphospholipid antibodies), inflammatory markers (complements, C-reactive protein, erythrocyte sedimentation rate), cardiac troponin I (cTnI), N-terminal proB-type natriuretic peptide (NT-proBNP), and renal function analysis. A standard 12-lead ECG was performed for all participants on admission, and data from Holter monitoring were also collected if available.

### 2.3. Cardiac Magnetic Resonance Imaging Analysis

Cardiovascular magnetic resonance images were acquired using a 3.0 T scanner (MAGNETOM Skyra, Siemens Healthineers, Erlangen, Germany). We obtained cine images via an electrocardiogram-gated two-dimensional balanced steady-state free precession sequence. LGE images were collected 10 min after the injection of 0.1 mmol/kg gadopentetate dimeglumine using a two-dimensional phase-sensitive inversion-recovery gradient-echo pulse sequence. Native and postcontrast T1 mapping was performed using a modified look-locker inversion recovery sequence in a four-chamber long-axis slice and apical, middle, and basal short-axis slices. T2 mapping was acquired using a T2-prepared steady-state free precession sequence. Cardiovascular magnetic resonance images were independently reviewed by experienced investigators who were blind to echocardiographic and clinical outcome data. The visual assessment of LGE was performed according to standardized postprocessing recommendations. The presence of LGE was defined as positive when focal myocardial enhancement was visible in both short-axis and matching long-axis views. Patients with only junctional LGE were classified in the LGE-negative group, as junctional LGE was shown to be nonpathological [18]. The locations of LGE, including the left ventricular free wall and septum, were recorded. LGE quantification, native T1, extracellular volume (ECV), and T2 were analyzed semiautomatically via cvi42 software Version 5.3 (Circle Cardiovascular Imaging, Calgary, AB, Canada). LGE quantification was performed by 2 senior operators using the full width at half maximum method. The degree of LGE was presented as a percentage and was calculated by dividing the LGE mass by the myocardial mass. LGE was also classified as mild, moderate, and severe based on equal tertiles of the LGE percentage (>0 and ≤1.94%, >1.94% and ≤4.94%, and >4.94%) [4]. The ECV was calculated using T1 of myocardium and T1 of the blood pool pre- and post-gadolinium contrast, along with the hematocrit value.

### 2.4. Echocardiography and Non-Invasive Pressure-Strain Loop Analysis

All echocardiographic examinations were performed using ultrasound machines GE Vivid 9 (GE Medical Systems, Waukesha, WI, USA). Two-dimensional dynamic images of at least three cardiac cycles from apical four-chamber, three-chamber, two-chamber, and long-axis views were acquired at a frame rate of 50 to 70 frames/second. Atrial and ventricular dimensions alongside volumes were measured according to the American Society of Echocardiography [19]. Apical four-chamber and two-chamber views were analyzed to evaluate LVEF via Simpson’s biplane method. Myocardial work was analyzed offline using specific software (EchoPAC Version 203; GE Medical Systems, USA). This non-invasive method utilizes brachial cuff blood pressure, which was assumed to be equivalent to left ventricular systolic pressure in conjunction with the longitudinal strain (LS) via speckle-tracking echocardiography. Arterial pressure was measured at rest immediately before the echocardiographic study. The LS was measured using apical four-chamber, three-chamber, and two-chamber views in the automated function imaging mode. The regional speckle area of interest was manually adjusted to obtain optimal tracking results. Global LS (GLS) was calculated using a 17-segment model at the time in systole when the value peaked. Meanwhile, PSD, which was the standard deviation of the time to peak strain in each segment, was obtained. Once GLS analysis was complete, the values of systolic blood pressure, which were assumed to be equal to the peak systolic left ventricular pressure, were introduced in the software, which constructed a non-invasive left ventricular pressure-strain loop adjusted according to the opening and closure of mitral and aortic valves. Myocardial work indices that were calculated included the myocardial work index (WI), constructive work (CW), wasted work (WW), and work efficiency (WE). WI was equivalent to the area of the pressure–strain loop. CW was defined as work during shortening in systole plus work during lengthening in isovolumetric ventricular contraction. WW was defined as work during lengthening in systole plus work during shortening in isovolumetric ventricular contraction. WE was the percentage of CW in the sum of CW and WW. Similar to LS, a Bull’s eye with segmental and global WI, CW, WW, and WE values were provided. During the regional analysis of strain and myocardial work, the values for 5 segments in the septum and 12 segments in the left ventricular free wall were averaged separately to obtain values at the corresponding regions.

### 2.5. Follow-Up

The clinical endpoint of this study included major adverse cardiac events (MACE), such as cardiac mortality, hospitalization for heart failure, and documented sustained ventricular arrhythmia (>30 s). All patients were followed up every 3–6 months on the telephone using the standard questionnaire interview and clinical medical records. The time until the endpoint was calculated from the date of the baseline cardiovascular magnetic resonance examination. The data of all patients were included up to the last date of follow-up.

### 2.6. Statistical Analysis

Continuous variables were expressed as the mean ± standard deviation (SD) or median with an interquartile range (IQR) based on a normal or skewed distribution. Categorical variables were presented as the frequency and percentages. Comparisons between 2 groups were carried out using the independent samples *t*-test or Mann–Whitney rank sum test, or chi-square test. Comparisons across 3 groups were performed using a one-way analysis of variance (ANOVA) or the Kruskal–Wallis test. Event-free survival was assessed using Kaplan–Meier analyses, with comparisons performed via the log-rank test. Univariate and multivariate Cox proportional hazard analyses were adopted to identify the predictors associated with prognosis. Initial univariate analyses were performed based on the primary parameters of interest, including clinical and imaging parameters. Subsequently, the variables with *p <* 0.05 in univariate analysis, as well as age, sex, and cTnI, were included in multivariate analysis. Repeated measurements of functional parameters were performed on 20 randomly selected participants. The intraclass correlation coefficient was applied for the assessment of intra-observer and inter-observer reliability. All statistical analyses were processed using a standard statistical software program (SPSS version 25.0; IBM Corp., Armonk, NY, USA). A *p*-value < 0.05 was considered statistically significant.

## 3. Results

### 3.1. Study Population and Clinical Characteristics

**Figure 1:**
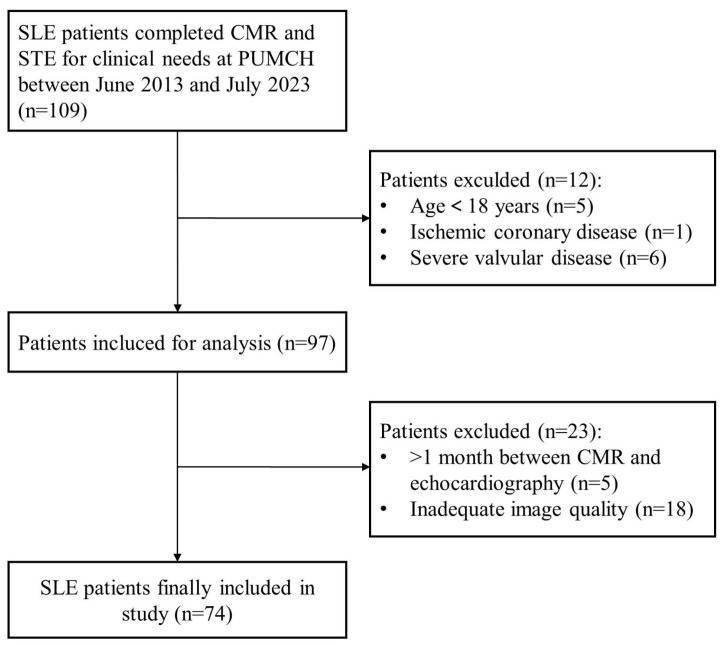
Study flowchart. The diagram describes the inclusion of SLE patients. Abbreviations: CMR, cardiac magnetic resonance; PUMCH, Peking Union Medical College Hospital; SLE, systemic lupus erythematosus; STE, speckle-tracking echocardiography.

A total of 109 SLE patients with clinically suspected myocardial involvement were screened. Of these, 35 patients were excluded due to age < 18 years (*n* = 5), ischemic coronary disease (*n* = 1), severe valvular disease (*n* = 6), more than 1 month between cardiovascular magnetic resonance and echocardiography (*n* = 5), and poor image quality (*n* = 18). Finally, 74 patients were enrolled in this study (Figure 1). A total of 37 patients with non-autoimmune diseases-related non-ischemic cardiomyopathy and 37 healthy controls were included. The baseline characteristics of the included 74 patients with SLE (mean age, 35 ± 12 years; 95% female) were shown in Table 1 and Table S1. Hypertension occurred in 22 (30%) patients, of which 9 (12%) had diabetes mellitus, and 7 (10%) had hyperlipidemia. Lupus nephritis was developed in 37 (50%) patients. The antiphospholipid syndrome was developed in 14 patients (19%), and 20 (27%) patients had pulmonary hypertension. The median NT-proBNP and cTnI were 840 pg/mL and 0.02 μg/L, respectively. Cardiac symptoms were present in 57 (77%) patients, including 18 (24%) patients with chest pain, 37 (50%) with dyspnea, 14 (19%) with palpitations, and 21 (28%) with edema.

### 3.2. Presence, Location, and Degree of LGE and Myocardial Dysfunction

Among the included 74 SLE patients, 39 (52.7%) developed LGE. The incidence of LGE in patients with SLE and patients with non-autoimmune diseases was comparable (52.7% vs. 67.6%, *p* = 0.135). In the context of comparable SLE-related disease activity and cumulative organ damage levels, SLE patients with LGE had serious cardiac symptoms and myocardial injury compared to those without LGE. SLE patients with LGE had a higher incidence of antiphospholipid syndrome than those without LGE. The global myocardial function was significantly impaired in all SLE patients regardless of the presence or absence of LGE when compared with the healthy controls (all *p* < 0.001). Meanwhile, SLE patients with LGE presented with lower global WE (GWE) than those without LGE (Table 2). For regional functional analysis, the left ventricular wall in each patient was divided into two regions according to the free wall and septum. The results indicate that regional functional parameters, including LS and WE, in regions with LGE were more impaired than those in regions without LGE (regional LS, −13.4 ± 4.4% vs. −15 ± 3.5%, *p* = 0.017; regional WE, 87.9 ± 6.6% vs. 90.7 ± 4.6%, *p* = 0.009).

**Figure 2:**
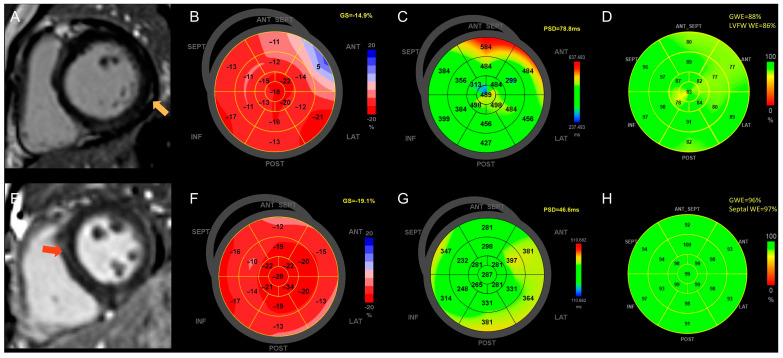
A SLE patient with LVFW LGE (**A**) (yellow arrow) had impaired GLS (**B**), increased PSD (**C**), decreased GWE, and decreased LVFW WE (**D**). A SLE patient with septal LGE (**E**) (red arrow) had relatively normal functional parameters (**F**–**H**). Abbreviations: GS, global longitudinal strain; GWE, global myocardial work efficiency; LVFW, left ventricular free-wall; PSD, peak strain dispersion; WE, myocardial work efficiency.

As for the location of LGE in SLE patients, LGE was present only in the septum of 20 (27%) cases, only in the left ventricular free wall in 9 (12%) cases, and in both locations in 10 (14%) cases. There was a difference in the location of LGE between patients with SLE and non-autoimmune diseases (*p* = 0.019). In patients with non-autoimmune disease-related cardiomyopathy, LGE is most often located in both the septum and free wall (*n* = 13, 35%), followed by only the septum (*n* = 8, 22%). Comparisons between patients with (*n* = 19) and without left ventricular free-wall (LVFW) LGE (*n* = 55) showed no significant difference in age, gender, and SLE disease activity (Table S2). In regional functional analysis, patients with LVFW LGE presented with worse regional functions, as evidenced by lower LVFW WE and higher LVFW WW compared with patients without LVFW LGE (Table S2). In global functional analysis, patients with LVFW LGE had a worse global myocardial function, represented by lower GWE, higher global WW, and higher global PSD in contrast to patients without LVFW LGE (Table S2). Moreover, comparisons between patients with septal-only LGE (*n* = 20) and LVFW-only LGE (*n* = 9) demonstrated that patients with LVFW-only LGE had higher global PSD when fibrosis degrees were comparable (Table S3). Figure 2 shows a SLE patient with LVFW-only LGE who presented with an impaired myocardial function, mainly in the form of decreased LVFW WE, diminished GWE, and increased PSD, while a SLE patient with septal-only LGE had a relatively normal myocardial function.

Patients with SLE had a comparable degree of LGE to patients with non-autoimmune disease-related cardiomyopathy [median (IQR): 1.74% (0%–3.84%) vs. 2.9% (0.8%–4.95%), *p* = 0.119]. In patients with SLE, the degree of LGE was associated with significant myocardial dysfunction, as represented by elevated PSD (*p* = 0.021) (Table S4).

All intraclass correlation coefficient values of GLS, PSD, GWE, global WI, global CW, and global WW were greater than 0.75, which indicated good intra-observer and inter-observer repeatability (Table S5).

### 3.3. Presence, Location, and Degree of LGE and Outcomes

Over a median follow-up of 25.5 (IQR 17-48.5) months, 32 MACEs occurred in 27 (36%) patients as follows: 11 (15%) cardiac deaths, 19 (26%) heart failure hospitalizations, and 2 (3%) cases of sustained ventricular arrhythmia. The incidence of MACE tended to be higher in patients with SLE compared to those with non-autoimmune diseases (37% vs. 19%, *p* = 0.058).

**Figure 3:**
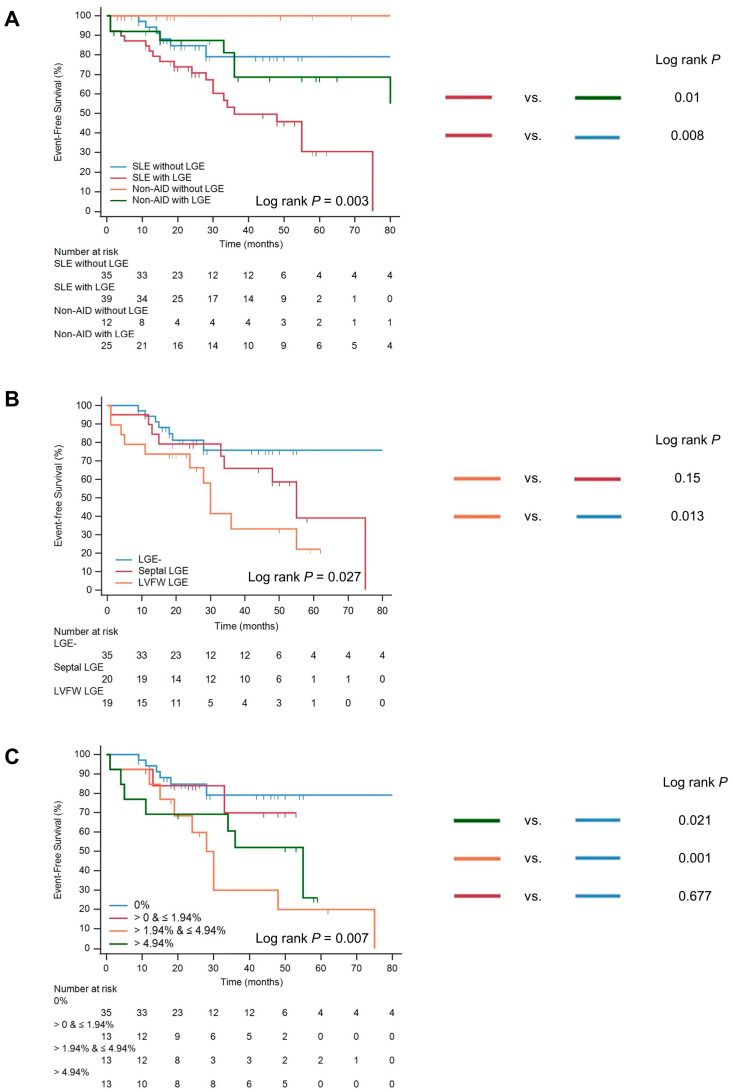
Kaplan–Meier curves for major adverse cardiac events in SLE patients. The differences in major adverse cardiac events were stratified by the presence of LGE (**A**), locations of LGE (**B**), and degrees of LGE (**C**). Abbreviations: AID, autoimmune diseases; LGE, late gadolinium enhancement; LVFW, left ventricular free-wall; SLE, systemic lupus erythematosus.

As for the association of the presence of LGE and outcomes, the Kaplan–Meier curve showed that SLE patients with LGE had worse outcomes compared with non-autoimmune disease patients with LGE (log-rank *p* = 0.01). Meanwhile, SLE patients with LGE had a higher incidence of MACE than those without LGE (log-rank *p* = 0.008) (Figure 3A). The univariable Cox analysis showed that the presence of LGE was associated with an increased risk of MACE (HR: 3.251, 95% CI: 1.295–8.159, *p* = 0.012) (Table 3). In the multivariable-adjusted model, the presence of LGE was an independent risk predictor of MACE (HR: 3.746, 95% CI: 1.434–9.79, *p* = 0.007) (Table 4).

Regarding the association of the location of LGE and outcomes, Kaplan–Meier analysis demonstrated that patients with LGE located in the left ventricular free wall had higher MACE rates compared with the other two groups with septal LGE and without LGE (overall log-rank *p* = 0.027) (Figure 3B). Univariable Cox analysis showed that LVFW LGE was associated with an increased risk of MACE (HR: 2.475, 95% CI: 1.134–5.399, *p* = 0.023) (Table 3). The multivariable Cox analysis demonstrated that LVFW LGE was an independent risk predictor of MACE (HR: 2.395, 95% CI: 1.023–5.606, *p* = 0.044) and cardiac mortality (HR: 6.349, 95% CI: 1.647–24.285, *p* = 0.007) (Table 4).

Regarding the association of the degree of LGE and outcomes, Kaplan–Meier analysis showed that the severe and moderate LGE group had higher incidences of MACE than the LGE-negative group (overall log-rank *p* = 0.007) (Figure 3C). The univariable and multivariable Cox analysis showed that severe and moderate LGE were associated with a high risk of MACE (moderate LGE HR: 5.258, 95% CI: 1.817–15.214, *p* = 0.002; severe LGE HR: 3.739, 95% CI: 1.241–11.266, *p* = 0.019) (Table 3 and Table 4).

The univariable and multivariable Cox analysis showed that, in addition to the different characteristics of LGE, the presence of SLE-related organ damage measured by SDI was significantly associated with MACE in SLE patients. When combining SLE-related organ damage and different characteristics of LGE to evaluate cardiac outcomes, SLE patients with both different characteristics of LGE and SLE-related organ damage were most likely to have predominantly shorter event-free survival (all log-rank *p* < 0.01) (Figure S1).

## 4. Discussion

**Figure 4:**
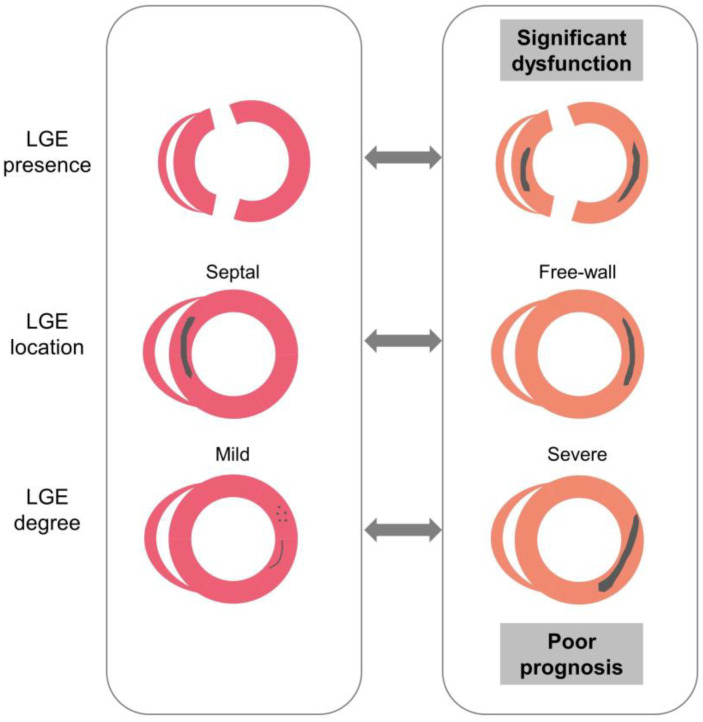
Our study shows how the presence, location, and degree of LGE were associated with myocardial dysfunction and poor prognosis in SLE patients. Abbreviations: LGE, late gadolinium enhancement; SLE, systemic lupus erythematosus.

In this retrospective study, we combined cardiovascular magnetic resonance and speckle-tracking echocardiography to evaluate myocardial injury in SLE patients. To our knowledge, this is the first study to examine the association between the presence, locations, and degrees of LGE and regional/global myocardial function and analyze their prognostic value in SLE patients. The main findings are as follows (Figure 4): First, the incidence and degree of myocardial fibrosis were comparable in patients with SLE and those with non-autoimmune diseases. The presence of fibrosis affected intrinsic myocardial function, and the effects varied by the degrees and locations of LGE in patients with SLE. Second, the presence, location, and degree of LGE were independently associated with poor cardiac outcomes in SLE patients. Finally, different characteristics of LGE combined with SLE-related systemic organ damage helped improve the risk stratification of patients with SLE.

Myocardial fibrosis is a histopathological remodeling process characterized by excessive accumulation in the myocardium of extracellular matrix components in response to an injury. LGE measured via cardiovascular magnetic resonance is a sensitive tool and a first-line non-invasive exam to detect myocardial fibrosis [5]. In our study, the incidence of LGE in the myocardium of SLE patients was 53%, and LGE was located more in the septum than in the free wall, which is consistent with previously reported results [20]. In SLE patients, the myocardium might be damaged either directly due to a localized autoimmune process involving the deposition of immune complexes and the activation of the complement system or indirectly via general chronic inflammation induced by other injured organs [21]. Myocardial fibrosis was reported to be more likely to develop in patients with SLE combined with an antiphospholipid syndrome in this study, which implicates the involvement of antiphospholipid antibodies in the pathogenesis of myocardial injury in SLE patients [22].

In recent years, several clinical studies have focused on the association between myocardial fibrosis and myocardial function. Patients with myocardial fibrosis have been discovered to manifest global myocardial dysfunction [11]. Some studies demonstrated how focal fibrosis is linked with regional dysfunction [23,24]. However, few studies have reported the relationship between myocardial function and fibrosis in SLE patients. Our study is the first to comprehensively analyze the relationship between different patterns of LGE and myocardial function. The results demonstrated that LGE affected regional and global myocardial function through different modalities. One potential explanation for the development of myocardial dysfunction due to fibrosis might be that fibrosis could interfere with the coordination of the myocardial excitation–contraction coupling. In addition, the disturbance of the fibrotic network could impair the transduction of cardiomyocyte contraction into myocardial force development, resulting in the uncoordinated contraction of cardiomyocyte bundles. Moreover, fibrosis destroyed the conduction and subsequent generation of reentry circuits, further impacting the overall function of the myocardium. Meanwhile, fibrosis might increase myocardial stiffness, leading to global or regional impairment in deformation [25].

Both LVEF and GLS are considered standard parameters for left ventricular quantification in guideline recommendations. However, both GLS and LVEF are susceptible to changes in preload and afterload, leading to the misinterpretation of the true systolic function of the myocardium. Since myocardial work incorporates both the strain and afterload simultaneously to reduce load dependence, myocardial work adds incremental value to the existing evaluation of the intrinsic myocardial function [26]. Our study found that myocardial work was significantly impaired in SLE patients with fibrosis when their LVEF and GLS were relatively normal, which indicated that myocardial work might have advantages in the early evaluation of myocardial injury in SLE. Abnormal myocardial work indices reflected the impairment of myocardial contractility and the disturbances of myocardial energy metabolism in SLE patients with myocardial fibrosis [8,9]. It has been reported that systolic synchrony was impaired in SLE patients [27]. Our study further reveals the possible pathologic mechanisms associated with elevated PSD. Various degrees and locations of focal fibrosis induced regional myocardial dysfunction, leading to the impairment of overall contractile synchrony.

Previous studies have shown that LGE is associated with an increased risk of adverse cardiac outcomes in non-ischemic cardiomyopathy [28,29]. Meanwhile, different patterns of LGE have been revealed to be important risk modifiers in myocardial diseases [30]. However, up until now, there has been a paucity of data examining the relationship between the presence, location, and degree of LGE and cardiac outcomes in SLE patients. Our data suggest that the presence of LGE is correlated with a worse cardiac prognosis in SLE patients, which might be secondary to myocardial dysfunction in relation to fibrosis [31]. Meanwhile, SLE patients with fibrosis were found to have a worse cardiac prognosis compared with non-autoimmune disease patients with fibrosis. SLE is characterized by the autoantibody formation and inflammation of multiple target organs. Chronic inflammation has been considered a key feature in cardiovascular disease pathogenesis in autoimmune diseases. SLE-related systemic organ damage, including but not limited to pulmonary arterial hypertension and renal disease, might affect cardiac prognosis [32,33]. In addition, patients with SLE were often exposed to immunomodulators and glucocorticoids. The long-term application of glucocorticoids might increase the risks of cardiovascular diseases [34]. Some immunosuppressive agents, such as antimalarial drugs, were found to be associated with myocardial injury [35].

Recent studies have identified the association between the location of LGE and clinical prognosis. Similar to this study, some studies showed that fibrosis located in the left ventricular free wall impacted the prognosis of the disease. A multicenter study of left ventricular noncompaction revealed that LVFW LGE was associated with an increased risk of MACE [14]. A cohort study of 557 patients with hypertrophic cardiomyopathy showed LVFW LGE as an independent predictor of adverse cardiac outcomes [36]. However, some studies of dilated cardiomyopathy emphasized the prognostic value of LGE located other than in the free wall. A cohort study of 874 patients with dilated cardiomyopathy identified that septal LGE was correlated with increased all-cause mortality, and sudden cardiac death was mostly linked with combined septal and free wall LGE [4]. Another retrospective study of 1165 patients with dilated cardiomyopathy demonstrated that combined septal and free-wall LGE was associated with a heightened risk of malignant arrhythmias [37]. Although these studies favored the clinical significance of septal fibrosis, the incremental prognostic value of combined free wall fibrosis was also confirmed in the above studies. The differences in locations of LGE between SLE patients and non-autoimmune disease patients were identified in this study, which might be related to the specificity of the effects of LGE locations on myocardial function in SLE. Our data showed that the location of LGE had an independent prognostic value in SLE patients, and LVFW LGE was associated with an increased risk of adverse cardiac outcomes. This variation in the impact of myocardium based on the location of fibrosis might be explained by various factors, including but not limited to differences in the etiological substrate, scar microstructure, and geographical effects. The activation of the immune system and chronic inflammation of SLE caused direct and indirect injuries to the myocardium. Different insults might trigger fibrosis with different microstructures. Additionally, the large proportion of free wall in the whole left ventricular myocardium might be a contributing factor to the different geographical effects observed.

The clinical significance of the degree of fibrosis in myocardial disease has also been the focus of recent studies of non-ischemic cardiomyopathy. The degree of LGE was found to be mainly associated with all-cause mortality [38] and MACE [39] in non-ischemic cardiomyopathies. Nevertheless, the definitions of different degrees of LGE were not consistent in different studies, which limited the translation of this technique into clinical practice [30]. This study demonstrated that the degree of LGE was correlated with myocardial dysfunction and adverse cardiac outcomes in SLE patients. Therefore, besides the presence of LGE, the location and degree might be far more clinically useful metrics to predict adverse cardiac outcomes in SLE patients. Further studies are needed to utilize different patterns of LGE to improve clinical prognosis in SLE patients.

The SDI has been widely utilized to assess cumulative organ damage in SLE. The SDI includes non-reversible changes in organs affected by the process of disease process. Our data showed that the SDI was an independent predictor of poor cardiac prognosis in SLE patients. Furthermore, patients with both cumulative organ damage and myocardial fibrosis had the highest risk of MACE. The results highlight that the combination of myocardial fibrosis and SLE-related systemic organ damage was conducive to identifying SLE patients at high risk for adverse clinical outcomes. Myocardial fibrosis had the potential to enhance the risk assessment and stratification of SLE patients.

Our study has several limitations. First, it was a single-center observational study with a limited cohort of SLE patients. However, the sample size in our research compared favorably with some previously published studies about myocardial injury by cardiovascular magnetic resonance in SLE [7]. Second, women made up the majority of included patients in this study. It is widely believed that SLE is more common in women than men, with women accounting for up to 93% of SLE patients, which is in accordance with gender distribution in the included patients of this study. Third, although patients with all levels of disease activity were included in this study, the proportion of patients with high disease activity was relatively high, limiting the generalization of our findings.

## 5. Conclusions

In SLE patients, focal fibrosis with different characteristics disturbed regional and global myocardial dysfunction. Their presence, location, and degree were independent predictors of poor prognosis in patients with SLE. Myocardial fibrosis combined with SLE-related organ damage helped to improve the cardiac-related risk stratification of SLE patients. The prevention and treatment of myocardial fibrosis need to be the focus of future research to improve the myocardial function and clinical prognosis of SLE patients.

## Figures and Tables

**Table 1:** Baseline clinical characteristics of SLE patients with and without LGE.

Variables	Total (N = 74)	LGE+ (N = 39)	LGE− (N = 35)	*p* Value
Age, years	35 ± 12	37 ± 13	32 ± 9	0.101
Female gender, *n* (%)	70 (95)	36 (92)	34 (97)	0.687
Hypertension, *n* (%)	22 (30)	12 (31)	10 (29)	0.836
Diabetes, *n* (%)	9 (12)	5 (13)	4 (11)	>0.999
Hyperlipidemia, *n* (%)	7 (10)	3 (8)	4 (11)	0.583
Smoking, *n* (%)	6 (8)	5 (13)	1 (3)	0.254
SLE detail				
Disease duration, months, median (IQR)	37 (3–99)	37 (4–96)	36 (2–120)	0.653
Antiphospholipid syndrome, *n* (%)	14 (19)	11 (28)	3 (9)	0.031
Pulmonary hypertension, *n* (%)	20 (27)	9 (23)	11 (31)	0.419
SLEDAI-2K score, median (IQR)	10 (6–18)	10 (5–19)	12 (6–19)	0.786
SDI, median (IQR)	1 (0–2)	1 (0–2)	1 (0–2)	0.448
Cardiac manifestation				
Cardiac symptom, *n* (%)	57 (77)	35 (90)	22 (63)	0.006
Arrhythmia, *n* (%)	21 (28)	14 (36)	7 (20)	0.13
NT-proBNP, pg/mL, median (IQR)	840 (251–3040)	2440 (601–6771)	385 (146–1119)	<0.001
cTnI, µg/L, median (IQR)	0.02 (0–0.18)	0.08 (0.01–0.47)	0.02 (0–0.04)	0.002
Medication				
Glucocorticoid, *n* (%)	69 (93)	35 (90)	34 (97)	0.422
Immunosuppressive agent, *n* (%)	68 (92)	33 (85)	35 (100)	0.046
Antiplatelet agents or anticoagulants, *n* (%)	22 (30)	17 (44)	5 (14)	0.006
ACEI/ARB, *n* (%)	28 (38)	18 (46)	10 (29)	0.119
Beta blockers, *n* (%)	34 (46)	23 (59)	11 (31)	0.018
MRA, *n* (%)	18 (24)	13 (33)	5 (14)	0.057

Abbreviations: ACEI, angiotensin-converting enzyme inhibitor; ARB, angiotensin receptor blocker; cTnI, cardiac troponin I; IQR, interquartile range; MRA, mineralocorticoid receptor antagonist; NT-proBNP, N-terminal proB-type natriuretic peptide; SDI, Systemic Lupus International Collaborating Clinics/American College of Rheumatology (SLICC/ACR) Damage Index; SLE, systemic lupus erythematosus; SLEDAI-2K, SLE Disease Activity Index 2000.

**Table 2:** Imaging parameters between SLE patients with and without LGE.

Variables	Healthy Controls (N = 37)	Total (N = 74)	LGE+ (N = 39)	LGE− (N = 35)	*p* Value
Conventional echocardiographic parameter					
IVSD, mm	8.9 ± 1.8	8.3 ± 1.6	8.9 ± 1.5	7.7 ± 1.6 *	0.003
LVPWD, mm	8.9 ± 1.8	8.4 ± 1.4	8.7 ± 1.3	8.0 ± 1.6 *	0.039
LVEDD, mm	43 ± 4	47 ± 6 *	47 ± 8 *	46 ± 5 *	0.477
LVESD, mm	28 ± 5	31 ± 7 *	32 ± 8 *	30 ± 5	0.136
LVEF, %	67 ± 7	61 ± 10 *	59 ± 11 *	64 ± 9	0.089
LVFS, %	38 ± 4	33 ± 7 *	32 ± 8 *	35 ± 6	0.039
E/A radio	1.6 ± 0.3	1.2 ± 0.4 *	1.2 ± 0.4 *	1.2 ± 0.4 *	0.979
LA diameter, mm	33 ± 3	33 ± 6	34 ± 6	32 ± 5	0.068
PASP, mmHg, median (IQR)	21 (19–24)	28 (23–56) *	28 (24–35) *	26 (22–43) *	0.419
TAPSE, mm	19 ± 2	18 ± 5 *	17 ± 4 *	19 ± 5	0.123
TRV, m/s	1.7 ± 0.5	2.6 ± 0.7 *	2.6 ± 0.7 *	2.6 ± 0.6 *	0.818
RV diameter, mm	22 ± 4	22 ± 4	22 ± 5	22 ± 3	0.606
STE parameter					
GWI, mmHg%	1750 ± 258	1323 ± 339 *	1289 ± 360 *	1360 ± 315 *	0.372
GCW, mmHg%	2113 ± 264	1609 ± 351 *	1572 ± 383 *	1650 ± 312 *	0.345
GWW, mmHg%, median (IQR)	67 (50–99)	143 (95–180) *	156 (117–178)*	119 (82–183) *	0.213
GWE, %	95.7 ± 1.8	90 ± 4.9 *	88.9 ± 5.4*	91.2 ± 4.1 *	0.047
GLS, %	−20.2 ± 2.0	−15.2 ± 3.4 *	−14.7 ± 3.8*	−15.8 ± 2.8 *	0.167
PSD, ms	37 ± 9	60 ± 19 *	64 ± 23*	56 ± 13 *	0.09
CMR parameter					
Native T1, ms	1266 ± 30	1388 ± 71 *	1392 ± 76 *	1383 ± 64 *	0.583
ECV, %	26 ± 2	33 ± 6 *	33 ± 6 *	33 ± 5 *	0.803
T2, ms	38 ± 1	42 ± 3 *	42 ± 3 *	42 ± 3 *	0.459

Abbreviations: ECV, extracellular volume fraction; GCW, left ventricular global constructive work; GLS, left ventricular global longitudinal strain; GWE, left ventricular global work efficiency; GWI, left ventricular global work index; GWW, left ventricular global wasted work; IQR, interquartile range; IVSD, inter-ventricular septum thickness at end-diastole; LA, left atrial; LGE, late gadolinium enhancement; LVEDD, left ventricular end diastolic diameter; LVEF, left ventricular ejection fraction; LVESD, left ventricular end systolic diameter; LVFS, left ventricular fraction shortening; LVPWD, left ventricular posterior wall thickness at end-diastole; PASP, systolic pulmonary artery pressure; PSD, left ventricular global peak strain dispersion; RV, right ventricular; SLE, systemic lupus erythematosus; TAPSE, tricuspid annular plane systolic excursion; TRV, tricuspid regurgitation velocity. * Significantly different (*p* < 0.05) compared with healthy controls.

**Table 3:** Univariable Cox regression of major adverse cardiac events in patients with SLE.

Variables	Univariable Analysis	
	HR (95% CI)	*p* Value
Clinical variables		
Age	1.021 (0.991–1.053)	0.169
Female sex	0.889 (0.120–6.607)	0.908
Hypertension	1.061 (0.459–2.451)	0.89
Diabetes	1.049 (0.246–4.480)	0.948
Hyperlipidemia	1.935 (0.719–5.203)	0.191
NYHA functional class	1.445 (0.973–2.146)	0.068
NT-proBNP, pg/mL	1.000 (1.000–1.000)	0.416
cTnI, µg/L	1.020 (0.877–1.187)	0.793
SLEDAI-2K score	0.991 (0.954–1.029)	0.624
SDI > 0	2.647 (1.147–6.106)	0.023
Glucocorticoid	1.124 (0.264–4.781)	0.874
Immunosuppressive treatment	1.320 (0.310–5.624)	0.708
Antiplatelet agents or anticoagulants	1.406 (0.59–3.349)	0.442
ACEI/ARB	1.322 (0.606–2.882)	0.483
Beta blockers	0.916 (0.429–1.958)	0.821
MRA	0.965 (0.407–2.288)	0.935
Imaging parameter		
LVEF, %	0.987 (0.952–1.023)	0.476
PASP, mmHg	1.000 (0.980–1.020)	0.978
GLS, %	1.041 (0.933–1.161)	0.474
PSD, ms	1.007 (0.988–1.027)	0.475
GWI, mmHg%	1.001 (1.000–1.002)	0.269
GCW, mmHg%	1.000 (0.999–1.002)	0.471
GWW, mmHg%	0.996 (0.989–1.002)	0.19
GWE, %	1.038 (0.950–1.134)	0.406
LGE presence	3.251 (1.295–8.159)	0.012
LGE degree, %	1.093 (1.019–1.172)	0.013
LGE degree (tertiles)		
>0 and ≤1.94%	1.499 (0.37–6.071)	0.57
>1.94% and ≤4.94%	4.725 (1.714–13.026)	0.003
>4.94%	3.282 (1.115–9.663)	0.031
LGE location		
LVFW LGE	2.475 (1.134–5.399)	0.023
Septal LGE	1.097 (0.499–2.411)	0.817
Native T1, ms	1.004 (0.999–1.009)	0.112
ECV, %	1.027 (0.963–1.095)	0.416
T2, ms	1.064 (0.928–1.220)	0.375

Abbreviations: ACEI, angiotensin-converting enzyme inhibitor; ARB, angiotensin receptor blocker; cTnI, cardiac troponin I; ECV, extracellular volume fraction; GCW, left ventricular global constructive work; GLS, left ventricular global longitudinal strain; GWE, left ventricular global work efficiency; GWI, left ventricular global work index; GWW, left ventricular global wasted work; LGE, late gadolinium enhancement; LVEF, left ventricular ejection fraction; LVFW, left ventricular free-wall; MRA, mineralocorticoid receptor antagonist; NT-proBNP, N-terminal proB-type natriuretic peptide; PASP, systolic pulmonary artery pressure; PSD, left ventricular global peak strain dispersion; SDI, Systemic Lupus International Collaborating Clinics/American College of Rheumatology (SLICC/ACR) Damage Index; SLE, systemic lupus erythematosus; SLEDAI-2K, SLE Disease Activity Index 2000.

**Table 4:** Multivariable Cox regression of major adverse cardiac events in patients with SLE.

Variables	LGE Presence Plus Covariables		LGE Location Plus Covariables		LGE Degree Plus Covariables	
	Adjusted HR (95% CI)	*p* Value	Adjusted HR (95% CI)	*p* Value	Adjusted HR (95% CI)	*p* Value
Age	0.999 (0.969–1.031)	0.97	1.003 (0.973–1.035)	0.826	0.998 (0.966–1.031)	0.901
Female sex	1.082 (0.141–8.3)	0.94	0.711 (0.093–5.465)	0.743	0.613 (0.07–5.36)	0.658
cTnI, µg/L	0.981 (0.842–1.143)	0.807	0.974 (0.834–1.138)	0.744	0.963 (0.82–1.13)	0.64
SDI > 0	3.125 (1.27–7.688)	0.013	2.515 (1.068–5.923)	0.035	2.828 (1.167–6.852)	0.021
LGE presence	3.746 (1.434–9.79)	0.007				
LVFW LGE			2.395 (1.023–5.606)	0.044		
LGE degree						
>0 and ≤1.94%					1.778 (0.405–7.798)	0.446
>1.94% and ≤4.94%					5.258 (1.817–15.214)	0.002
>4.94%					3.739 (1.241–11.266)	0.019

Variables with *p* < 0.05 in univariate Cox analysis, as well as age, sex, and cTnI, were constructed as multivariable Cox regression models with LGE presence, location, and degree, respectively. Abbreviations: cTnI, cardiac troponin I; LGE, late gadolinium enhancement; LVFW LGE, left ventricular free-wall late gadolinium enhancement; SDI, Systemic Lupus International Collaborating Clinics/American College of Rheumatology (SLICC/ACR) Damage Index; SLE, systemic lupus erythematosus.

## Data Availability

All data relevant to this study are included in the article.

## Supplementary Materials

The following supporting information can be downloaded at: https://www.mdpi.com/article/10.3390/jcdd10110451/s1, Figure S1: Kaplan–Meier curves for major adverse cardiac events; Table S1: Cardiac manifestations and SLE details of patients with SLE; Table S2: Clinical and imaging parameters between SLE patients with and without LVFW LGE; Table S3: Clinical and imaging parameters of SLE patients with only septal versus only free wall fibrosis; Table S4: The association between myocardial function and the degree of LGE in SLE patients; Table S5: Intra- and inter-observer variability for parameters in the non-invasive LV PSL analysis.
