# Supplementary material for: The Presence, Location, and Degree of Late Gadolinium Enhancement in Relation to Myocardial Dysfunction and Poor Prognosis in Patients with Systemic Lupus Erythematosus
Source: J Cardiovasc Dev Dis. 2023 Oct 31;10(11):451. doi: 10.3390/jcdd10110451 (PMC10672496; doi:10.3390/jcdd10110451)
Supplement: Supplementary file 1 [file jcdd-10-00451-s001.zip › jcdd-2638988-supplementary.pdf]

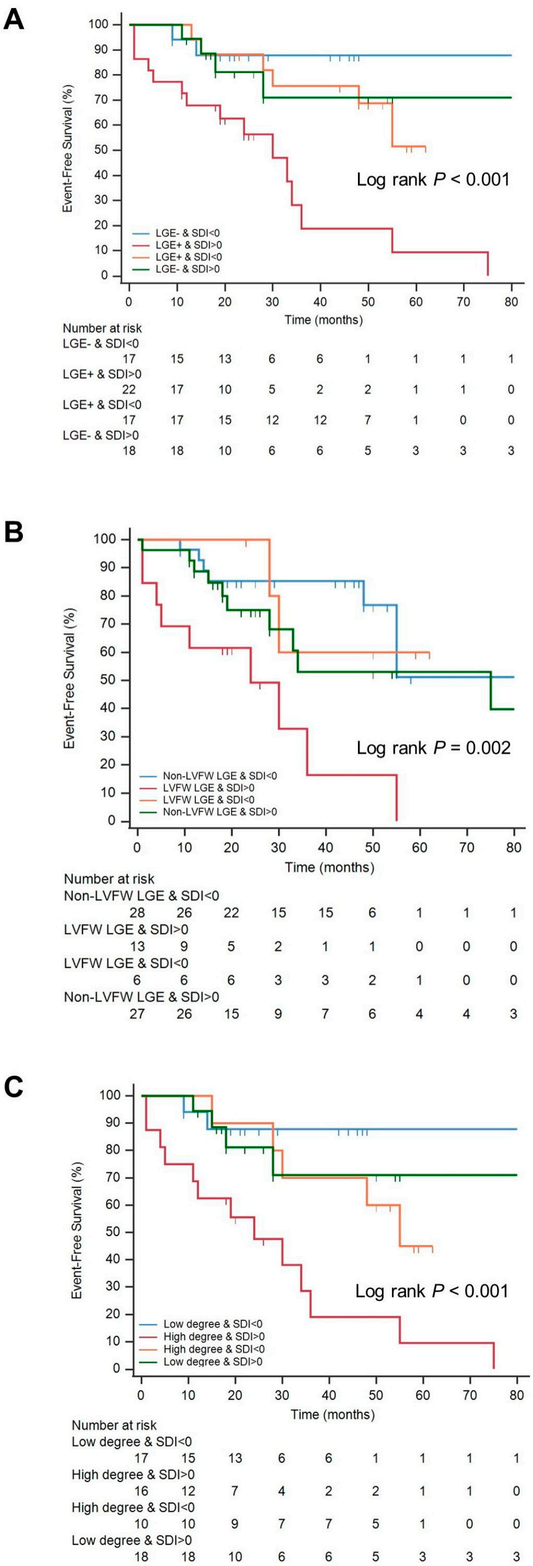


**Figure S1.** Kaplan–Meier curves for major adverse cardiac events. The differences in major adverse cardiac events were stratified by SLE related organ damage (measured by SDI > 0) combined with LGE presence (A), locations of LGE (B), and degrees of LGE (C), respectively. Abbreviations: LGE, late gadolinium enhancement; LVFW, left ventricular free-wall; SDI, the Systemic Lupus International Collaborating Clinics/American College of Rheumatology (SLICC/ACR) Damage Index; SLE, systemic lupus erythematosus.

**Table S1.** Cardiac manifestations and SLE details of patients with SLE.

| Variables | Total (N=74) | LGE + (N=39) | LGE - (N=35) | *p* Value |
|---|---|---|---|---|
| **Cardiac manifestation** | | | | |
| SBP, mmHg | 116±13 | 116±13 | 116±13 | 0.802 |
| DBP, mmHg | 75±11 | 77±11 | 73±12 | 0.195 |
| HR, beats/min | 84±12 | 85±11 | 83±14 | 0.506 |
| Cardiac symptom, n (%) | 57 (77) | 35 (90) | 22 (63) | 0.006 |
| Chest pain, n (%) | 18 (24) | 12 (31) | 6 (17) | 0.173 |
| Dyspnea, n (%) | 37 (50) | 24 (62) | 13 (37) | 0.036 |
| Palpitation, n (%) | 14 (19) | 7 (18) | 7 (20) | 0.822 |
| Edema, n (%) | 21 (28) | 11 (28) | 10 (29) | 0.972 |
| Arrhythmia, n (%) | 21 (28) | 14 (36) | 7 (20) | 0.13 |
| Ventricular arrhythmia, n (%) | 10 (14) | 5 (13) | 5 (14) | 0.854 |
| Atrial arrhythmia, n (%) | 9 (12) | 5 (13) | 4 (12) | >0.999 |
| Conduction block, n (%) | 3 (4) | 2 (5) | 1 (3) | >0.999 |
| pericardial effusion, n (%) | 36 (49) | 20 (51) | 16 (46) | 0.632 |
| LDL-C, mmol/L, median (IQR) | 2.4 (1.9-3.2) | 2.52 (2.03-3.34) | 2.355 (1.783-2.885) | 0.442 |
| **SLE detail** | | | | |
| Mucocutaneous manifestations, n (%) | 37 (50) | 18 (46) | 19 (54) | 0.485 |
| Musculoskeletal manifestations, n (%) | 27 (37) | 12 (31) | 15 (43) | 0.281 |
| NPSLE, n (%) | 15 (20) | 7 (18) | 8 (23) | 0.6 |
| Lupus nephritis, n (%) | 37 (50) | 19 (49) | 18 (52) | 0.816 |
| Serum C3, g/l, median (IQR) | 0.69 (0.35-0.85) | 0.705 (0.431-0.952) | 0.601 (0.329-0.845) | 0.559 |
| Serum C4, g/l, median (IQR) | 0.10 (0.03-0.15) | 0.098 (0.039-0.147) | 0.055 (0.016-0.146) | 0.158 |
| CRP, mg/l, median (IQR) | 2.9 (1.0-12.7) | 3.6 (0.56-12.5) | 2.76 (1.02-11.89) | 0.733 |
| ESR, mm/h, median (IQR) | 31 (14-59) | 29 (14-56) | 23 (14-62) | 0.653 |
| Anti-nuclear antibody, n (%) | 70 (95) | 36 (93) | 34 (97) | 0.687 |
| Anti-double-stranded DNA, n (%) | 49 (66) | 23 (59) | 26 (74) | 0.164 |
| Anti-Smith antibodies, n (%) | 24 (32) | 12 (31) | 12 (34) | 0.747 |
| Anticardiolipin antibody, n (%) | 15 (20) | 10 (26) | 5 (14) | 0.225 |
| Anti-β2-glycoprotein 1 antibodies, n (%) | 16 (22) | 10 (26) | 6 (17) | 0.375 |
| Lupus anticoagulant, n (%) | 22 (30) | 13 (33) | 9 (26) | 0.474 |
| 24-hour urine protein, g, median (IQR) | 0.2 (0.1-1.0) | 0.23 (0.108-1.003) | 0.205 (0.118-1.593) | 0.726 |
| Serum creatinine, umol/L, median (IQR) | 63 (50-83) | 64 (50-99) | 61 (50-75) | 0.279 |
| D-Dimer, mg/L FEU, median (IQR) | 1.7 (0.6-3.8) | 1.82 (0.49-3.62) | 1.66 (0.93-3.99) | 0.548 |

Abbreviations: CRP, C-reactive protein; DBP, diastolic blood pressure; ESR: erythrocyte sedimentation rate; HR, heart rate; IQR, interquartile range; LDL-C, low-density lipoprotein cholesterol; LGE, late gadolinium enhancement; LVFW, left ventricular free-wall; NPSLE: neuropsychiatric lupus; SBP, systolic blood pressure; SLE, systemic lupus erythematosus.

**Table S2.** Clinical and imaging parameters between SLE patients with and without LVFW LGE.

| Variables | LVFW LGE (N=19) | No LVFW LGE (N=55) | *p* Value |
|---|---|---|---|
| **Clinical variables** | | | |
| Age, years | 39±15 | 33±10 | 0.094 |
| Female gender, n (%) | 18 (95) | 52 (95) | >0.999 |
| SLEDAI-2K score, median (IQR) | 13 (6-22) | 10 (5-18) | 0.638 |
| SDI, median (IQR) | 2 (0-3) | 0 (0-1) | 0.024 |
| NT-proBNP, pg/mL, median (IQR) | 2699 (835-6779) | 643 (186-2592) | 0.002 |
| cTnI, ug/L, median (IQR) | 0.25 (0.02-0.76) | 0.02 (0-0.10) | 0.004 |
| **Conventional echocardiographic parameter** | | | |
| IVSD, mm | 9.7±1.3 | 7.9±1.5 | <0.001 |
| LVPWD, mm | 9.2±1.3 | 8.1±1.4 | 0.005 |
| LVEDD, mm | 48±7 | 46±6 | 0.402 |
| LVESD, mm | 33±7 | 31±6 | 0.226 |
| LVEF, % | 59±11 | 62±10 | 0.245 |
| LVFS, % | 32±8 | 34±7 | 0.204 |
| E/A radio | 1.3±0.5 | 1.2±0.3 | 0.838 |
| LA diameter, mm | 37±6 | 32±5 | 0.003 |
| PASP, mmHg, median (IQR) | 29 (24-36) | 27 (22-38) | 0.267 |
| TAPSE, mm | 17±5 | 18±5 | 0.336 |
| TRV, m/s | 2.7±0.8 | 2.6±0.6 | 0.364 |
| RV diameter, mm | 23±4 | 22±5 | 0.458 |
| **STE parameter** | | | |
| GWI, mmHg% | 1210±304 | 1362±344 | 0.091 |
| LVFW WI, mmHg% | 1215±331 | 1394±372 | 0.068 |
| Septal WI, mmHg% | 1198±288 | 1298±315 | 0.227 |
| GCW, mmHg% | 1499±320 | 1647±356 | 0.112 |
| LVFW CW, mmHg% | 1485±336 | 1673±379 | 0.06 |
| Septal CW, mmHg% | 1526±359 | 1597±350 | 0.455 |
| GWW, mmHg%, median (IQR) | 156 (138-178) | 125 (82-181) | 0.043 |
| LVFW WW, mmHg%, median (IQR) | 152 (131-193) | 109 (82-185) | 0.02 |
| Septal WW, mmHg%, median (IQR) | 165 (124-193) | 150 (79-186) | 0.301 |
| GWE, % | 87.4±4.6 | 90.9±4.8 | 0.007 |
| LVFW WE, % | 87.1±6.2 | 91.4±4.5 | 0.002 |
| Septal WE, % | 87.9±4.4 | 89.8±6.0 | 0.203 |
| GLS, % | -14.1±3.3 | -15.6±3.4 | 0.09 |
| LVFW LS, % | -13.4±4.6 | -15.7±3.9 | 0.037 |
| Septal LS, % | -12.9±3.5 | -14.2±3.4 | 0.174 |
| PSD, ms | 74±25 | 55±14 | 0.005 |
| **CMR parameter** | | | |
| Native T1, ms | 1408±83 | 1381±65 | 0.171 |
| ECV, % | 34±7 | 32±5 | 0.21 |
| T2, ms | 43±3 | 42±3 | 0.514 |

Abbreviations: cTnI, cardiac troponin I; CW, constructive work; ECV, extracellular volume fraction; GCW, left ventricular global constructive work; GLS, left ventricular global longitudinal strain; GWE, left ventricular global work efficiency; GWI, left ventricular global work index; GWW, left ventricular global wasted work; IVSD, inter-ventricular septum thickness at end-diastole; LA, left atrial; LGE, late gadolinium enhancement; LS, longitudinal strain; LVEDD, left ventricular end diastolic diameter; LVEF, left ventricular ejection fraction; LVESD, left ventricular end systolic

diameter; LVFS, left ventricular fraction shortening; LVFW, left ventricular free-wall; LVPWD, the left ventricular posterior wall thickness at end-diastole; NT-proBNP, N-terminal proB-type natriuretic peptide; PASP, systolic pulmonary artery pressure; PSD, left ventricular global peak strain dispersion; RV, right ventricular; SDI, Systemic Lupus International Collaborating Clinics/American College of Rheumatology (SLICC/ACR) Damage Index; SLE, systemic lupus erythematosus; SLEDAI-2K, SLE Disease Activity Index 2000; TAPSE, tricuspid annular plane systolic excursion; TRV, tricuspid regurgitation velocity; WE, work efficiency; WI, work index; WW, wasted work.

**Table S3.** Clinical and imaging parameters of SLE patients with only septal versus only free-wall fibrosis.

| Variables | Only Septal LGE (N=20) | Only LVFW LGE (N=9) | *p* Value |
|---|---|---|---|
| **Conventional echocardiographic parameter** | | | |
| IVSD, mm | 8.1±1.3 | 9.4±0.7 | 0.005 |
| LVPWD, mm | 8.3±1.1 | 9.0±1.1 | 0.106 |
| LVEDD, mm | 46±8 | 48±6 | 0.634 |
| LVESD, mm | 32±8 | 33±5 | 0.729 |
| LVEF, % | 60±11 | 59±8 | 0.853 |
| LVFS, % | 32±7 | 31±6 | 0.827 |
| E/A radio | 1.2±0.3 | 1.2±0.5 | 0.901 |
| LA diameter, mm | 32±6 | 36±6 | 0.133 |
| PASP, mmHg, median (IQR) | 28 (23-34) | 28 (24-31) | 0.813 |
| TAPSE, mm | 17±4 | 18±5 | 0.699 |
| TRV, m/s | 2.5±0.6 | 2.4±0.3 | 0.65 |
| RV diameter, mm | 22±6 | 21±4 | 0.697 |
| **STE parameter** | | | |
| GWI, mmHg% | 1365±398 | 1303±335 | 0.689 |
| LVFW WI, mmHg% | 1393±440 | 1315±387 | 0.649 |
| Septal WI, mmHg% | 1309±350 | 1281±263 | 0.828 |
| GCW, mmHg% | 1643±430 | 1583±379 | 0.725 |
| LVFW CW, mmHg% | 1667±465 | 1560±422 | 0.562 |
| Septal CW, mmHg% | 1594±404 | 1629±382 | 0.826 |
| GWW, mmHg%, median (IQR) | 136 (80-178) | 156 (150-170) | 0.211 |
| LVFW WW, mmHg%, median (IQR) | 130 (76-180) | 157 (133-191) | 0.12 |
| Septal WW, mmHg%, median (IQR) | 146 (75-224) | 169 (118-193) | 0.572 |
| GWE, % | 90.4±5.9 | 88.4±4.2 | 0.388 |
| LVFW WE, % | 90.8±5.4 | 87.8±7.1 | 0.226 |
| Septal WE, % | 89.4±7.6 | 89.6±3.3 | 0.953 |
| GLS, % | -15.3±4.2 | -14.9±3.4 | 0.806 |
| LVFW LS, % | -15.2±5.0 | -13.9±5.6 | 0.552 |
| Septal LS, % | -14.1±4.3 | -13.9±2.4 | 0.898 |
| PSD, ms | 53±16 | 79±29 | 0.036 |
| **CMR parameter** | | | |
| Native T1, ms | 1378±69 | 1414±97 | 0.283 |
| ECV, % | 31±5 | 35±5 | 0.108 |
| T2, ms | 41±2 | 43±3 | 0.292 |
| LGE degree, %, median (IQR) | 2.05 (1.24-4.86) | 3.82 (1.58-5.52) | 0.322 |

Abbreviations are shown in the table S2.

**Table S4.** The association between myocardial function and the degree of LGE in SLE patients.

| Variables | >0% & ≤ 1.94% (N=13) | > 1.94% & ≤ 4.94% (N=13) | > 4.94% (N=13) | *p* Value |
|---|---|---|---|---|
| LVEF, % | 59±11 | 62±13 | 57±10 | 0.597 |
| PSD, ms | 52±11 | 66±29 | 72±22 | 0.021 |
| GLS, % | -14.5±3.6 | -15.4±3.6 | -14.3±4.4 | 0.756 |
| LVFW LS, % | -14.3±4.3 | -15.2±4.5 | -13.5±5.8 | 0.674 |
| Septal LS, % | -14.4±4.6 | -13.3±3.1 | -13.0±4.0 | 0.656 |
| GWI, mmHg% | 1290±339 | 1318±382 | 1260±384 | 0.922 |
| LVFW WI, mmHg% | 1303±374 | 1343±421 | 1273±420 | 0.906 |
| Septal WI, mmHg% | 1263±318 | 1269±336 | 1234±336 | 0.959 |
| GCW, mmHg% | 1568±387 | 1559±423 | 1590±366 | 0.979 |
| LVFW CW, mmHg% | 1569±422 | 1570±459 | 1595±387 | 0.985 |
| Septal CW, mmHg% | 1565±363 | 1538±405 | 1580±397 | 0.961 |
| GWW, mmHg%, median (IQR) | 148 (78-168) | 154 (117-187) | 167 (132-186) | 0.507 |
| LVFW WW, mmHg%, median (IQR) | 141 (84-161) | 132 (85-200) | 152 (130-205) | 0.254 |
| Septal WW, mmHg%, median (IQR) | 154 (87.665-224) | 165 (90-221) | 145 (106-193) | 0.921 |
| GWE, % | 90.2±5.5 | 88.0±6.2 | 88.5±4.7 | 0.562 |
| LVFW WE, % | 126±47 | 141±64 | 177±70 | 0.103 |
| Septal WE, % | 158±86 | 158±65 | 158±89 | >0.999 |

Abbreviations: CW, constructive work; GCW, left ventricular global constructive work; GLS, left ventricular global longitudinal strain; GWE, left ventricular global work efficiency; GWI, left ventricular global work index; GWW, left ventricular global wasted work; IQR, interquartile range; LGE, late gadolinium enhancement; LS, longitudinal strain; LVEF, left ventricular ejection fraction; LVFW, left ventricular free wall; PSD, left ventricular global peak strain dispersion; SLE, systemic lupus erythematosus; WE, work efficiency; WI, work index; WW, wasted work.

**Table S5.** Intra- and inter-observer variability for parameters in the noninvasive LV PSL analysis.

| Variables | Intraobserver variability | | Interobserver variability | |
|---|---|---|---|---|
| | **ICC (95% CI)** | ***p* Value** | **ICC (95% CI)** | ***p* Value** |
| GLS | 0.984 (0.974, 0.99) | <0.001 | 0.976 (0.958, 0.986) | <0.001 |
| PSD | 0.997 (0.993, 0.998) | <0.001 | 0.992 (0.971, 0.997) | <0.001 |
| GWI | 0.943 (0.909, 0.965) | <0.001 | 0.924 (0.879, 0.953) | <0.001 |
| GCW | 0.954 (0.916, 0.974) | <0.001 | 0.965 (0.942, 0.979) | <0.001 |
| GWW | 0.988 (0.977, 0.994) | <0.001 | 0.980 (0.967, 0.988) | <0.001 |
| GWE | 0.989 (0.98, 0.994) | <0.001 | 0.973 (0.933, 0.987) | <0.001 |

Abbreviations: GCW, left ventricular global constructive work; GLS, left ventricular global longitudinal strain; GWE, left ventricular global work efficiency; GWI, left ventricular global work index; GWW, left ventricular global wasted work; ICC, intraclass correlation coefficient; LV, left ventricular; PSD, left ventricular global peak strain dispersion; PSL, pressure-strain loop.
